# Chalcone-based Pyrazoline Derivatives as Modulators of Neuroinflammatory and Redox Pathways in Experimental Epilepsy

**DOI:** 10.1007/s12035-026-05795-y

**Published:** 2026-03-18

**Authors:** Elif Azize Özşahin Delibaş, Zeynep Kasap Acungil, Esra Koç, Şeyma Özsoy

**Affiliations:** 1 Faculty of Health Sciences, Department of Nutrition and Dietetics, Tokat Gaziosmapasa University, 60250 Tokat, Turkey; 2 Faculty of Health Sciences, Department of Physiotherapy and Rehabilitation, Tokat Gaziosmapasa University, 60250 Tokat, Turkey; 3 Faculty of Arts and Sciences, Department of Chemistry, Tokat Gaziosmapasa University, 60250 Tokat, Turkey; 4 Faculty of Medicine, Department of Physiology, Tokat Gaziosmapasa University, 60250 Tokat, Turkey

**Keywords:** Chalcone, COX-2, 5-LOX, KEAP1- NRF2, Neuroinflammation, Oxidative Stress

## Abstract

Epilepsy is a chronic neurological condition characterized by complex pathophysiological interactions involving oxidative stress (OS) and neuroinflammation. In this study, the neuroprotective effects of chalcone-based pyrazoline derivatives (CCA-326, CCA-334, CCD-326, and CCD-334) are investigated in a penicillin-induced epilepsy model in rats. The ELISE method is used to measure the effects of these compounds on the plasma and brain levels of cyclooxygenase-2 (COX-2), 5-lipoxygenase (5-LOX), nuclear factor-kappa B (NF-κB), Kelch-like ECH-associated protein 1 (KEAP1), and nuclear factor erythroid 2-related factor 2 (NRF2). Total antioxidant status (TAS) and total oxidant status (TOS) were determined using colorimetric methods. CCA-326 and CCA-334 significantly reduces NF-κB, COX-2, and 5-LOX levels, increases NRF2 expression and TAS levels, and supports the activation of the endogenous antioxidant system by inducing a noticeable decrease in KEAP1 levels. These results suggest that CCA-334 stands out as a promising compound for further investigation targeting oxidative stress and inflammation in epilepsy, and that chalcone-based pyrazoline derivatives may modulate key inflammatory and redox pathways involved in epileptogenesis.

**Graphical Abstract:**

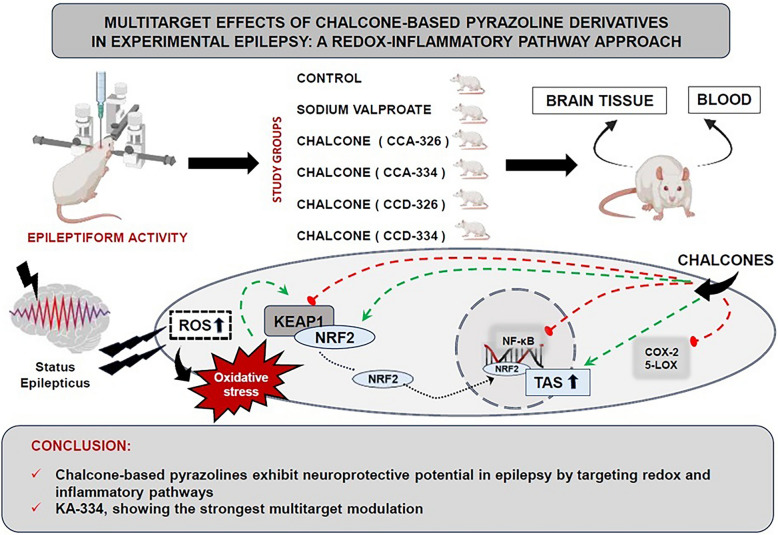

**Supplementary Information:**

The online version contains supplementary material available at 10.1007/s12035-026-05795-y.

## Introduction

Epilepsy is a chronic neurological disorder that involves recurrent seizures, affects millions of people worldwide, and can lead to severe consequences in the cognitive, psychological, and social domains [1, 2]. Despite current anticonvulsant drugs, many patients experience uncontrollable seizures or resistance to treatment, which points to a clinical need for new and more effective treatments with different effect mechanisms [3].

Epileptogenesis is modulated by a multicomponent and complex molecular network that is shared by the interaction of oxidative stress (OS) and inflammatory signals [1, 4]. These two mechanisms in the disease pathophysiology cause neuronal damage and seizure induction by creating a reciprocal cycle, in which they trigger each other [5–7]. Elevated production of reactive oxygen species (ROS) and inadequate antioxidant defense during epileptic activity cause both structural and functional disruptions [5], and the neuroinflammation caused by the accompanying proinflammatory cytokine secretion and activation of microglia and astrocyte reinforces the development of epilepsy through neuronal hyperexcitability [6]. In this pathophysiological process, double-action molecules that both suppress OS and limit inflammatory response by simultaneously modulating the two mechanisms offer a promising potential for epilepsy treatment [8].

Pyrazoline derivatives are significant pharmacophoric structures that play a role in redox balance and the regulation of inflammatory mediators [9, 10]. Several chalcone and pyrazoline derivatives were previously reported to have potent anti-inflammatory and antioxidant activities. For instance, cardamonin, a natural chalcone, exhibits neuroprotective effects through NF-κB inhibition and Nrf2 activation [11], while 4,5-dihydro-1H-pyrazole derivatives bearing aromatic substituents offers COX-2 inhibition and redox modulation [12]. Among these, two major scaffolds (carboxamide and carbothioamide-based pyrazolines) stand out thanks to their distinct pharmacological profiles. Carboxamide structures are known for their higher metabolic stability and potential for interaction with COX/LOX enzymes through hydrogen bonding and lipophilic interactions [12], whereas carbothioamides, with sulfur-based moieties, may participate in thiol-mediated antioxidant mechanisms [9, 10]. These structural differences may lead to differential biological activity, a hypothesis investigated through comparative analysis in the present study. Pyrazoline derivatives that contain halogen and methoxy groups exhibit stronger antioxidant and anti-inflammatory activities. Similarly, natural or semisynthetic chalcones can suppress these processes by targeting inflammatory enzymes due to their structural properties [11, 13]. Obtained from the reaction of chalcones with hydrazine derivatives, chalcone-based pyrazoline compounds have antiepileptic, anti-inflammatory, and neuroprotective potential. Previous studies indicate that these structures can act as multi-target agents that suppress the proconvulsive process [13, 14].

The cyclooxygenase (COX) and 5-lipoxygenase (5-LOX) enzymes represent different branches of arachidonic acid metabolism, and they moderate the formation of proinflammatory mediators. While cyclooxygenase-2 (COX-2) is stimulated particularly during inflammation, the use of COX inhibitors may elevate the leukotriene synthesis by shifting the flow of arachidonic acid to the 5-LOX pathway; it may lead to the continuation of inflammation or adverse effects. Thus, dual inhibitors that target COX-2 and 5-LOX together can provide a more balanced and effective anti-inflammatory response [15, 16]. Nuclear Factor kappa-light-chain-enhancer of activated B cells (NF-κB) raise the activation of target genes such as tumor necrosis factor-alpha (TNF-α), COX-2, and inducible nitric oxide synthase (iNOS) since they are activated through the joint effect of OS, proinflammatory cytokines, and neurotoxic signals, whereas chalcone and pyrazoline derivatives can suppress inflammatory response by inhibiting this pathway [13, 17, 18]. The bidirectional interaction between NF-κB and ROS creates a vicious cycle that exacerbates neuronal damage in epileptogenesis [17], while the Kelch-like ECH-associated protein 1/Nuclear factor erythroid 2-related factor 2/Antioxidant Response Element (KEAP1/NRF2/ARE) pathway balances this process by activating antioxidant defense [6]. Previous studies suggest that antioxidant agents developed by targeting the KEAP1/NRF2 pathway offer a complementary potential in epilepsy treatment, and that the reduction of COX-5 and 5-LOX expression by the inhibition of the NF-κB pathway has critical therapeutic importance [6, 17].

This study investigates the effects of chalcone-based pyrazoline derivatives (CCA-326, CCA-334, CCD-326, and CCD-334) on the COX-2, 5-LOX, NF-κB, KEAP1, and NRF2 pathways in an epilepsy model. The simultaneous modulation of inflammation and oxidative stress, which play a determining role in epileptogenesis, was targeted, and it was demonstrated by using a comprehensive approach that chalcone derivatives could offer a therapeutic potential by affecting multiple molecular pathways.

## Material and Method

Tokat Gaziosmanpaşa University’s Animal Experiments Local Ethics Committee (2025 HADYEK-05) approved the protocol of this study, which was conducted by following the principles specified in the European Union Directive (2010/63/EU).

### Design and Synthesis of Chalcone-Based Pyrazoles

Structural confirmation of synthesized compounds was assigned by elemental analyses, ^1^H-, ^13^C NMR and IR spectral data. All the reagents and the solvents used were pure at analytical grade. The reactions were followed by thin layer chromatography (TLC) on Merck 60 F254 (0.2 mm), silica gel preprepared plates. Melting points were measured on Electrothermal 9100 apparatus. IR spectrums (KBr or liquid) were recorded on a Jasco FT/IR-430 spectrometer. 1H and ^13^C NMR spectra were recorded using a Brucker Avance III instrument (400 MHz). As internal standards served TMS (d 0.00) for ^1^H-NMR and CDCl_3_ (d 77.0) for ^13^C NMR spectroscopy J values are given in Hz. The multiplicities of the signals in the 1H-NMR spectra are abbreviated by s (singlet), d (doublet), t (triplet), q (quartet), m (multiplet), br (broad), and their combinations.

#### Synthesis of Chalcones

**Fig. 1:**
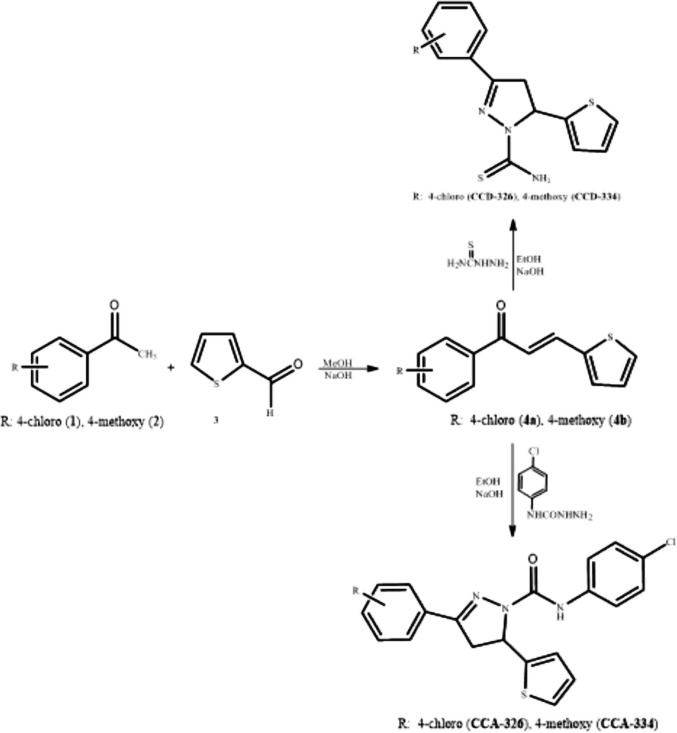
Synthesis of chalcone-based pyrazoles

Chalcones were synthesized via the Claisen-Schmidth condensation reaction of thiophene-2-carbaldehyde and acetophenone derivatives [19]. 2.5 M of aqueous NaOH solution was added to a solution of thiophene-2-carbaldehyde (0.1 mol) and ketone (0.1 mol) (4-chloroacetophenone, 4-methoxy acetophenone) in methanol, and the mixture was stirred at room temperature for 3 h. At the end of the reaction, the methanol was removed utilizing an evaporator, and the residue was dissolved in CHCl_3_, first neutralized with 5% HCl, and then extracted by washing with water. After drying the extract over Na_2_SO_4_, the solvent was removed. The obtained solid product was crystallized from ethanol (Fig. 1).

*1-(4-chlorophenyl)−3-(thiophen-2-yl)prop-2-en-1-one.* 95%, m.p. 101 °C. ^1^H-NMR (400 MHz, CDCl3, ppm): δ = 8.04 (s, 1H), 7.72–7.70 (m, 2H), 7.50–7.44 (m, 3H), 7.28–7.21 (m, 4H), 7.11–7.08 (m, 1H), 6.97–6.95 (m, 1H), 5.95–5.90 (m, 1H), 3.86–3.78 (m, 1H), 3.41–3.36 (m, 1H). ^13^C-NMR (100 MHz, CDCl3, ppm): δ = 151.20, 148.37, 141.73, 141.70, 136.94, 136.52, 135.64, 129.16, 128.90, 127.79, 127.00, 124.92, 120.27, 119.61, 50.66, 42.71. IR (KBr, cm^−1^): 3106, 3083, 3053, 2200, 1654, 1582, 1558, 1392, 1323, 1278, 1210, 1087,1026, 1008, 970, 814, 738, 670, 537, 457.

*1-(4-methoxyphenyl)−3-(thiophen-2-yl)prop-2-en-1-one.* 97%, m.p. 100 °C. ^1^H-NMR (400 MHz, CDCl_3_, ppm): δ = 8.04 (s, 1H), 7.72–7.70 (d, J = 8 Hz, 2H), 7.50–7.44 (d, J = 8 Hz, 2H), 7.28–7.24 (m, 2H), 7.22–7.20 (m, 1H), 7.10–7.09 (m, 1H), 7.00–6.94 (m, 1H), 5.90–5.86 (m, 1H), 3.89 (s, 3H), 3.85–3.77 (m, 1H), 3.41–3.35 (m, 1H). ^13^C-NMR (100 MHz, CDCl_3_, ppm): δ = 161.10, 152,22, 151.45, 137.17, 128.86, 128.22, 127.75, 126.93, 124.75, 124.68, 123.56, 120.20, 114.28, 55.76, 55.46, 43.00. IR (KBr, cm^−1^): 3099, 3079, 3015, 2969, 2932, 2836, 1650, 1586, 1566, 1361, 1251, 1171, 1106,1026, 1008, 973, 829, 807, 707, 677, 566, 498.

#### Synthesis of Carboxamides (CCA-326, CCA-334)

**Fig. 2:**
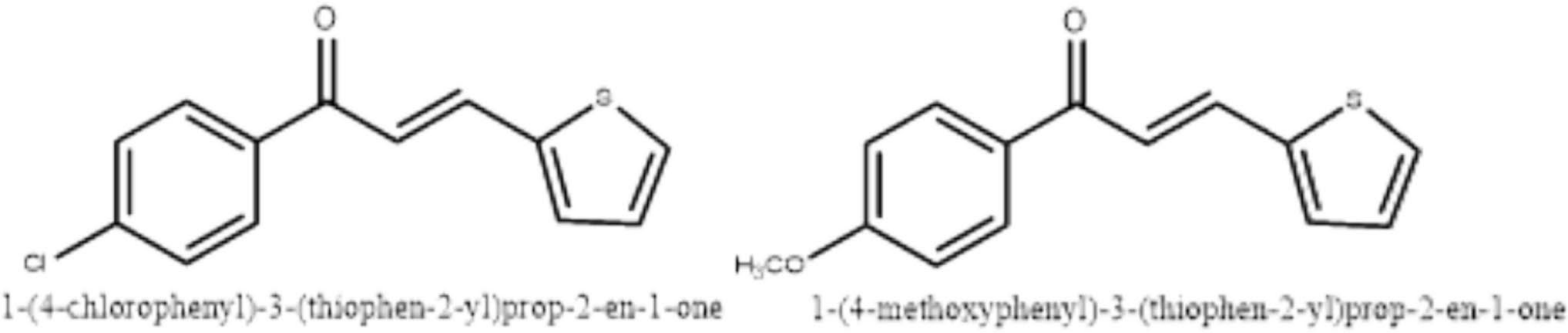
Structure of the synthesized chalcones

4-chlorophenyl isocyanate (5 mmol) was dissolved in diethyl ether. Hydrazine (5 mmol) was added dropwise to the mixture and stirred for 15 min. The resulting 4-chlorophenyl semi-carbazides were filtered, dried, and washed with petroleum ether. Chalcone (2 mmol) was dissolved in ethanol (10 mL), and then semi-carbazide was added. Finally, 1 ml of NaOH solution (0.02 M) was introduced. Upon the completion of the reaction, the mixture was poured onto ice, and the precipitate was filtered, dried, and recrystallized from a suitable solvent [20] (Fig. 1, Fig. 2).

*CCA-326.* 83%, m.p. 187 °C. ^1^H-NMR (400 MHz, CDCl_3_, ppm): δ = 7.74–7.71 (d, J = 12 Hz, 2H), 7.08–7.07 (d, J = 4 Hz, 1H), 7.01–6.98 (m, 1H), 6.98–6.94 (m, 3H), 6.39–6.35 (m, 1H), 3.90 (m, 3H), 3.76–3.80 (m, 1H), 3.40–3.35 (m, 1H). ^13^C-NMR (100 MHz, CDCl3, ppm): δ = 151.20, 148.37, 141.73, 141.70, 136.94, 136.52, 135.64, 129.16, 128.90, 127.79, 127.00, 124.92, 120.27, 119.61, 50.66, 42.71. IR (KBr, cm^−1^): 3384, 3102, 3007, 2977, 2916, 1673, 1593, 1517, 1400, 1376, 1312, 1274, 1228, 1167, 1122, 1069, 1008, 821, 799, 730, 711, 593, 525, 506, 441, 430.

*CCA-334*. 85%, m.p. 174 °C. ^1^H-NMR (400 MHz, CDCl_3_, ppm): δ = 7.74–7.71 (d, J = 12 Hz, 2H), 7.08–7.07 (d, J = 4 Hz, 1H), 7.01–6.98 (m, 1H), 6.98–6.94 (m, 3H), 6.39–6.35 (m, 1H), 3.88 (m, 3H), 3.77–3.82 (m, 1H), 3.42–3.36 (m, 1H). ^13^C-NMR (100 MHz, CDCl_3_, ppm): δ = 151.20, 148.37, 141.73, 141.70, 136.94, 136.52, 135.64, 129.16, 128.90, 127.79, 127.00, 124.92, 120.27, 119.61, 50.66, 42.71. IR (KBr, cm^−1^): 3384, 3102, 3007, 2977, 2916, 1673, 1593, 1517, 1400, 1376, 1312, 1274, 1228, 1167, 1122, 1069, 1008, 821, 799, 730, 711, 593, 525, 506, 441, 430.

#### Synthesis of Carbodiimides (CCD-326, CCD-334)

Chalcones (0.01 M) were dissolved in methanol. Thiosemicarbazide (0.01 M) was added to the solution. NaOH (0.02 M) was then introduced, and the mixture was refluxed under a condenser for 12 h. After the reaction was completed, the solution was cooled, diluted with water, and acidified using concentrated HCl. The product was then filtered, dried, and recrystallized from ethanol [20] (Fig. 1, Fig. 2).

*CCD-326.* 85%, m.p. 189 °C. ^1^H-NMR (400 MHz, CDCl_3_, ppm): δ = 7.72–7.69 (d, J = 12 Hz, 2H), 7.45–7.42 (d, J = 12 Hz, 2H), 7.21 (m, 1H), 7.08 (m, 1H), 6.96 (m, 1H), 6.42–6.38 (m, 1H), 3.85–3.77 (m, 1H), 3.40–3.35 (m, 1H). ^13^C-NMR (100 MHz, CDCl_3_, ppm): δ = 176.75, 155.00, 143.84, 137.25, 129.25, 129.04, 128.20, 126.82, 125.07, 124.71, 59.29, 42.81. IR (KBr, cm^−1^): 3475, 3338, 3132, 3083, 3065, 1566, 1453, 1353, 1335, 1300, 1259, 1194, 1080, 1004, 814, 700, 635, 529, 517, 430.

*CCD-334.* 89%, m.p. 157 °C. ^1^H-NMR (400 MHz, CDCl_3_, ppm): δ = 7.74–7.71 (d, J = 12 Hz, 2H), 7.08–7.07 (d, J = 4 Hz, 1H), 7.01–6.98 (m, 1H), 6.98–6.94 (m, 3H), 6.39–6.35 (m, 1H), 3.90 (m, 3H), 3.76–3.80 (m, 1H), 3.40–3.35 (m, 1H). ^13^C-NMR (100 MHz, CDCl_3_, ppm): δ = 176.23, 162.03, 156.09, 144.13, 128.73, 126.75, 124.95, 124.58, 123.05, 114.35, 59.05, 55.49, 42.94. IR (KBr, cm^−1^): 3406, 3243, 3140, 2965, 2938, 2893, 1582, 1563, 1468, 1414, 1373, 1304, 1236, 1171, 1073, 1026, 1012, 909, 836, 810, 711, 673, 601, 487.

**Fig. 3:**
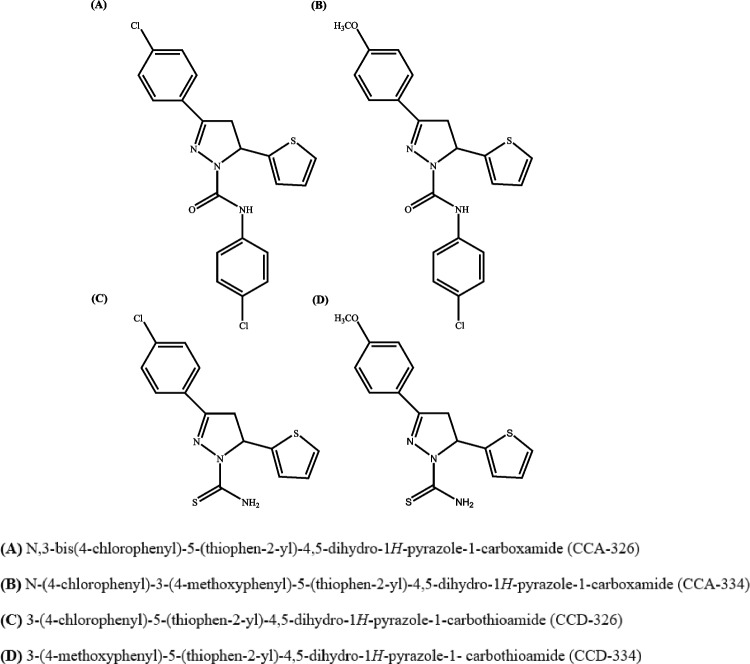
Structures of chalcone-based pyrazoline derivatives

The synthetic profiles and chemical structures of the compounds are presented in Fig. 3 and Table 1. Chalcone-based compounds were synthesized at a high yield via Claisen-Schmidt condensation, following the method reported in the literature [14].

**Table 1:** Substituent Patterns of Synthesized Chalcone-Based Pyrazole Derivatives

Code	Core scaffold	Position-1 substituent	Position-3 substituent	Position-5 substituent
CCA-326	4,5-dihydro-1H-pyrazol-1-carboxamide	N-(4-chlorophenyl)	4-chlorophenyl	thiophen-2-yl
CCA-334	4,5-dihydro-1H-pyrazol-1-carboxamide	N-(4-chlorophenyl)	4-methoxyphenyl	thiophen-2-yl
CCD-326	4,5-dihydro-1H-pyrazol-1-carbothioamide	—	4-chlorophenyl	thiophen-2-yl
CCD-334	4,5-dihydro-1H-pyrazol-1-carbothioamide	—	4-methoxyphenyl	thiophen-2-yl

### Experimental Animals and Groups

A total of 36 male *Wistar albino* rats, aged 12–16 weeks and weighing 200 ± 50 g, were used in this study. All animals were housed in plastic cages under standard laboratory conditions with a 12-h light/12-h dark cycle at a temperature of 23 ± 2 °C. There was unrestricted access to food and water. Animals that did not meet the experimental research standards were excluded. Groups were formed using randomly selected animals, with six rats assigned to each group.

The groups and treatment protocols were as follows:

1. Penicillin group (PG): 500 IU penicillin (2.5 µL, intracortical) [21, 22].
2. VPA group (SVG): 500 IU penicillin (2.5 µL, i.c.) + 300 mg/kg valproic acid (VPA, intraperitoneal) [23, 24].
3. Chalcone group (CCA-326): 500 IU penicillin (2.5 µL, i.c.) + 100 mg/kg CCA-326 (i.p.) [25].
4. Chalcone group (CCA-334): 500 IU penicillin (2.5 µL, i.c.) + 100 mg/kg CCA-334 (i.p.).
5. Chalcone group (CCD-326): 500 IU penicillin (2.5 µL, i.c.) + 100 mg/kg CCD-326 (i.p.).
6. Chalcone group (CCD-334): 500 IU penicillin (2.5 µL, i.c.) + 100 mg/kg CCD-334 (i.p.).

The doses used for each treatment group (penicillin, VPA, and chalcone derivatives) were selected by reviewing the previous in vivo studies reporting pharmacological efficacy in similar models [21–25]. In particular, 100 mg/kg was chosen for chalcone-based compounds as reported by Choudhary et al. [25].

### Experimental Procedure

The rats were anesthetized via the i.p. administration of 1.25 g/kg (25%) urethane and then restrained in a stereotaxic apparatus. A rostro-caudal incision with an approximately 3 cm length was made, and 500 IU of penicillin (Penicillin-G, 2.5 µL, i.c.) was injected into the left neocortex to induce epileptiform activity. The injection was performed using a Hamilton microinjector (710SNR) at a rate of 0.5 µL/min, targeting coordinates 3 mm lateral, 2 mm posterior, and 2 mm ventral from the bregma, at a depth of 1.5 mm.

Epileptiform activity was observed within 1–2 min following penicillin administration and reached a stable level within approximately 20–30 min. At this point, no further intervention was applied to PG, while SVG received 300 mg/kg sodium valproate (i.p.), and the chalcone groups were administered 100 mg/kg of CCA-326, CCA-334, CCD-326, or CCD-334 (i.p.). Intracardiac blood samples were collected from anesthetized rats 180 min after the administration, followed by decapitation and the extraction of brain tissue. Brain samples were rinsed with cold saline to remove residual blood, and they were then frozen for biochemical analysis. Blood samples were centrifuged at 3000 g for 15 min at + 4 °C, and plasma samples were stored at −80 °C until analysis.

### Tissue Homogenization and Biochemical Analyses

Brain tissue samples were rinsed three times with cold saline and dried using filter paper. Fresh tissue samples were weighed on a precision scale and homogenized at a 1:9 ratio in phosphate-buffered saline (PBS; Sigma P4417, Lot: #SLCH5832; pH 7.2–7.6) over ice using a Teflon-tipped homogenizer (Bandelin). The homogenates were centrifuged at 5000 g for 5 min at + 4 °C, aliquoted, and stored at −80 °C until the day of analysis.

Plasma and tissue homogenates were thawed for biochemical analyses. Protein concentrations were determined using the Thermo Scientific™ Pierce™ BCA Protein Assay Kit (Catalog Nos: 23225 and 23227).

COX-2, 5-LOX, NF-κB, KEAP1, and NRF2 levels in plasma and tissue samples were quantified using ELISA kits from BT LAB (Bioassay Technology Laboratory, #202 5/F 2 Bldg, 501 Changsheng S Rd, Nanhu Dist, Jiaxing, Zhejiang, China), with the following catalog numbers, respectively: E0296Ra, E0027Ra, E0287Ra, E2124Ra, and E1083Ra. Total antioxidant status (TAS) and total oxidant status (TOS) were determined colorimetrically using kits procured from Rel Assay Diagnostics (Gaziantep, Türkiye). All kit procedures were carried out by following the instructions of their manufacturers.

### *In Silico *Analyses

#### *In Silico* Molecular Docking Analysis

Docking analyses were conducted using a blind docking approach to identify potential binding cavities and plausible ligand orientations. Protein structures were retrieved from the Protein Data Bank and prepared before docking. The resulting docking poses are presented as supportive *in silico* observations in the Supplementary Material (Supplementary Figures S1 and S2).

#### *In Silico* ADMET Prediction

*In silico* ADMET prediction analyses were conducted for all synthesized chalcone-based pyrazoline derivatives using the SwissADME web server to obtain preliminary insights into their pharmacokinetic properties. The resulting predicted ADMET profiles are presented in the Supplementary Material (Supplementary Table S1).

### Statistical Analysis

Biochemical measurement data were analyzed using the SPSS software (version 20.0). The normality of the data distribution was tested using the Shapiro–Wilk test. For parameters exhibiting normal distribution, intergroup comparisons were performed using one-way ANOVA. Scheffe’s post hoc test was applied when significant differences were found. The Kruskal–Wallis test was used for non-normally distributed parameters, and pairwise group comparisons were conducted using the Mann–Whitney U test. The results are presented as mean ± standard error of the mean (SEM), and the threshold for statistical significance was set at p ≤ 0.05 for all analyses.

## Results

In this study, plasma and brain tissue samples collected from rats in a penicillin-induced experimental epilepsy model were analyzed using ELISA and colorimetric methods to determine the levels of oxidative stress markers (KEAP1, NRF2, TAS, and TOS) and inflammation markers (NF-κB, COX-2, 5-LOX). The levels of NF-κB, COX-2, 5-LOX, KEAP1, and NRF2, along with TAS and TOS, were compared across the CCA-326, CCA-334, CCD-326, and CCD-334 groups in relation to PG and SVG (Tables 2, 3 and 4).

**Table 2:** Changes in plasma and tissue NF-κB, COX-2 and 5-LOX levels in all groups. A significant decrease was observed in both plasma and tissue, particularly in CCA 326 and CCA 334, in comparison to PG

GROUPS	NF-κB		COX-2		5-LOX	
	Plasma (ng/mL)	Tissue (ng/mg protein)	Plasma (pg/mL)	Tissue (pg/mg protein)	Plasma (ng/mL)	Tissue (ng/g protein)
PG	7.94 ± 0.54	1.84 ± 0.39	4052.02 ± 1725.59	4697.62 ± 890.43	10.19 ± 0.66	9.38 ± 0.74
SVG	5.60 ± 0.21^•^	0.88 ± 0.38^•^	2161.29 ± 667.59^•^	858.19 ± 123.76^•^	8.49 ± 0.74^•^	6.00 ± 0.85^•^
CCA 326	3.68 ± 0.63^•†^	0.38 ± 0.09^•†a^	1813.93 ± 560.56^•a^	1224.61 ± 395.29^•a^	6.35 ± 0.74^•†^	6.13 ± 0.35^•ab^
CCA 334	3.73 ± 0.31^•†^	0.39 ± 0.09^•†a^	1586.33 ± 215.12^•a^	762.54 ± 194.41^•a^	7.89 ± 1.11^•^	5.36 ± 0.98^•a^
CCD 326	4.24 ± 0.71^•^	0.83 ± 0.06^•b^	3378.91 ± 434.67^b^	2740.32 ± 639.32^•†b^	6.63 ± 0.78^•†^	6.73 ± 0.63^•bc^
CCD 334	4.88 ± 0.71^•^	1.14 ± 0.25^•b^	3480.10 ± 308.12^b^	2556.39 ± 1287.19^a^	9.11 ± 2.34	7.56 ± 0.66^•†c^
p	*	*	*	*	*	*

*p ≤ 0.001, significant difference in plasma and tissue

± : SEM, •: vs to PG, †: vs to SVG, different superscript letters (a, b, c) within the same column indicate statistically significant differences between chalcone groups

*PG* Penicillin Group, *SVG* Sodium Valproat Group, *CCA-326* Chalcone Group, *CCA* 334 Chalcone Group, *CCD* 326 Chalcone Group, *CCD* 334 Chalcone Group

*NF-κB* Nuclear Factor kappa-light-chain-enhancer of activated B cells, *COX-2* Cyclooxygenase-2, 5-LOX: 5-lipoxygenase

**Table 3:** Changes in plasma and tissue KEAP 1 and NRF 2 levels in all groups. A significant decrease in KEAP1 levels and an increase in NRF levels were observed in both plasma and tissue in the CCA 334 and CCD 334 groups in comparison to the PG group

GROUPS	KEAP 1		NRF 2	
	Plasma (ng/L)	Tissue (ng/g protein)	Plasma (ng/mL)	Tissue (ng/g protein)
PG	2214.49 ± 130.04	482.50 ± 80.67	10.52 ± 0.82	4.38 ± 0.43
SVG	1200.69 ± 131.53^•^	293.62 ± 66.33^•^	12.09 ± 0.38^•^	6.86 ± 0.84^•^
CCA 326	1093.64 ± 178.01^•a^	389.05 ± 52.76^a^	12.57 ± 0.76^•a^	6.37 ± 0.54^•ab^
CCA 334	606.63 ± 71.68^•†b^	298.46 ± 35.90^•ab^	16.44 ± 0.99^•†b^	6.86 ± 0.69^•a^
CCD 326	1005.00 ± 48.35^•ac^	377.28 ± 50.19^ab^	14.11 ± 0.36^•†c^	5.29 ± 0.63^†b^
CCD 334	838.22 ± 111.49^•†c^	281.27 ± 27.00^•b^	15.38 ± 0.71^•†b^	5.82 ± 0.64^•ab^
p	*	*	*	*

*p ≤ 0.001, significant difference in plasma and tissue ± : SEM, •: vs to PG, †: vs to SVG, different superscript letters (a, b, c) within the same column indicate statistically significant differences between chalcone groups

*PG* Penicillin Group, *SVG* Sodium Valproat Group, *CCA-326* Chalcone Group, *CCA* 334 Chalcone Group, *CCD* 326: Chalcone Group, *CCD* 334 Chalcone Group

*KEAP 1* Kelch-like ECH-associated protein 1, *NRF 2* Nuclear factor erythroid 2–related factor 2

**Table 4:** Changes in plasma and tissue TAS and TOS in all groups. A significant decrease in TOS levels and an increase in TAS levels were observed in plasma in the CCA 334 and CCD 334 groups in comparison to the PG group

GROUPS	TAS		TOS	
	Plasma (mmol Trolox Eq/L)	Tissue (mmol Trolox Eq/mg protein)	Plasma (umol H_2_O_2_ Eq/L)	Tissue (umol H_2_O_2_ Eq/mg protein)
PG	1.12 ± 0.04	1.06 ± 0.13	7.29 ± 1.01	11.04 ± 1.00
SVG	1.54 ± 0.15^•^	1.28 ± 0.18	7.93 ± 0.66	9.22 ± 0.80
CCA 326	1.52 ± 0.07^•^	1.13 ± 0.35	4.07 ± 0.36^•†^	10.05 ± 0.79^a^
CCA 334	1.58 ± 0.16^•^	1.34 ± 0.40	3.19 ± 0.32^•†^	7.10 ± 0.61^•†b^
CCD 326	1.50 ± 0.09^•^	1.23 ± 0.36	4.10 ± 0.59^•†^	9.99 ± 0.66^a^
CCD 334	1.56 ± 0.12^•^	1.30 ± 0.32	3.82 ± 0.58^•†^	8.08 ± 0.27^•b^
p	*	#	*	*

# *p* > 0.05, non-significant difference

*p ≤ 0.001, significant difference in plasma and tissue ± : SEM, •: vs to PG, †: vs to SVG, different superscript letters (a, b, c) within the same column indicate statistically significant differences between chalcone groups

*PG* Penicillin Group, *SVG* Sodium Valproat Group, *CCA-326* Chalcone Group, *CCA* 334 Chalcone Group, *CCD* 326 Chalcone Group, *CCD* 334 Chalcone Group

*TAS* Total Antioxidant Status, *TOS* Total Oxidant Status

### Inflammatory Markers

**Fig. 4:**
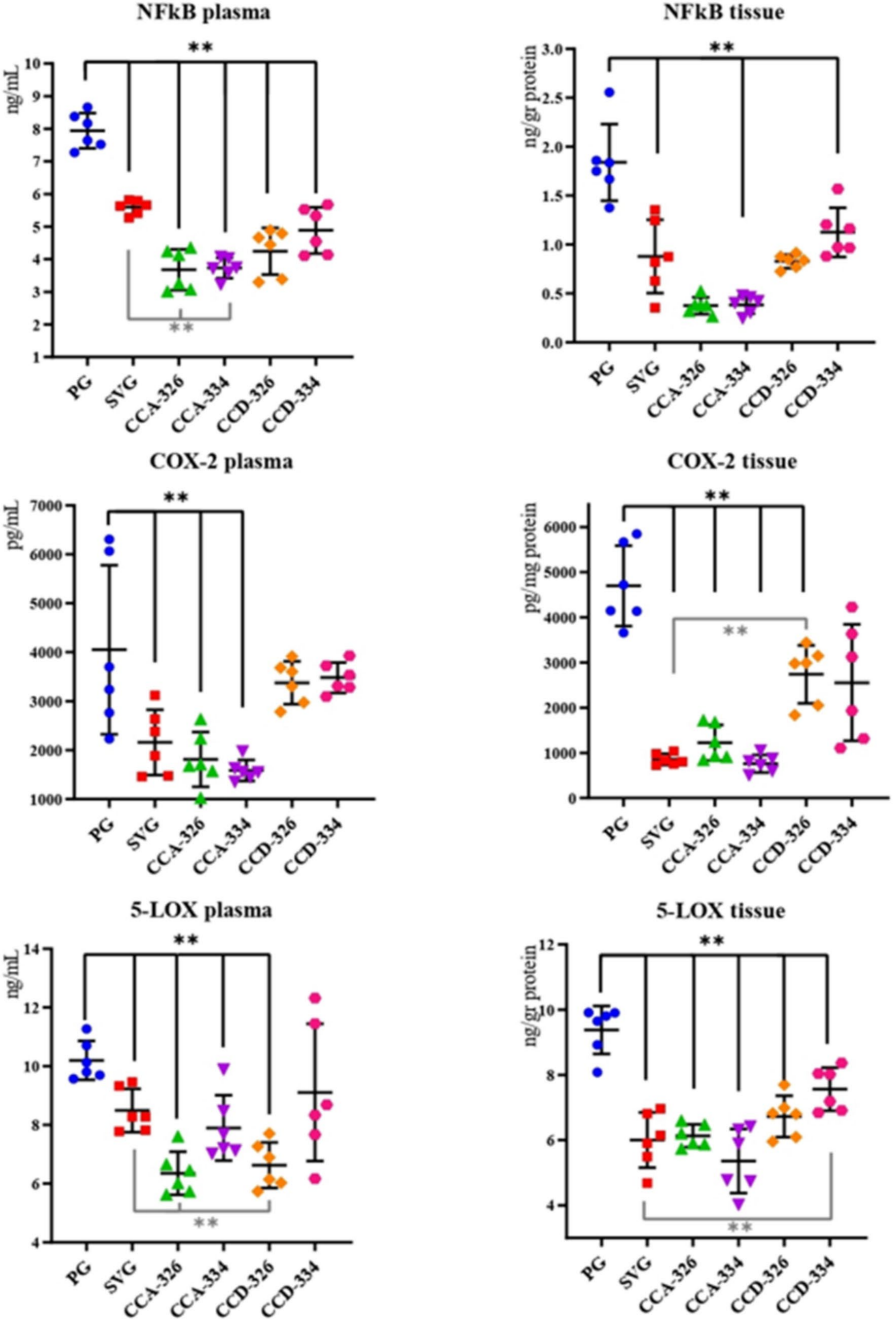
Changes in plasma and tissue NF-kB, COX-2, and 5-LOX levels by groups

In the CCA-326 and CCA-334 groups, plasma and tissue NF-κB levels were significantly lower than in PG and SVG (*p* < 0.001). The reduction in tissue NF-κB levels in CCA-326 and CCA-334 was also significant compared to the CCD groups (*p* < 0.001). COX-2 levels in both plasma and tissue samples were significantly lower in the CCA-326 and CCA-334 groups compared to PG, SVG, and the CCD groups (*p* < 0.001). Plasma 5-LOX levels were significantly reduced in CCA-326, CCA-334, CCD-326, and SVG relative to PG (*p* < 0.001). The reductions in CCA-326 and CCD-326 were also significant compared to SVG (†). Tissue 5-LOX levels were significantly lower in SVG, CCA-326, CCA-334, CCD-326, and CCD-334 groups compared to PG (*p* < 0.001). Among the chalcone groups, CCA-334 yielded the lowest tissue 5-LOX levels, and CCD-334 exhibited the highest tissue 5-LOX levels (Table 2, Fig. 4).

In summary, NF-κB, COX-2, and 5-LOX levels in both plasma and tissue samples were significantly lower in the CCA-326 and CCA-334 groups compared to all other groups (*p* < 0.001) (Table 2, Fig. 4).

### Oxidative Stress Markers

**Fig. 5:**
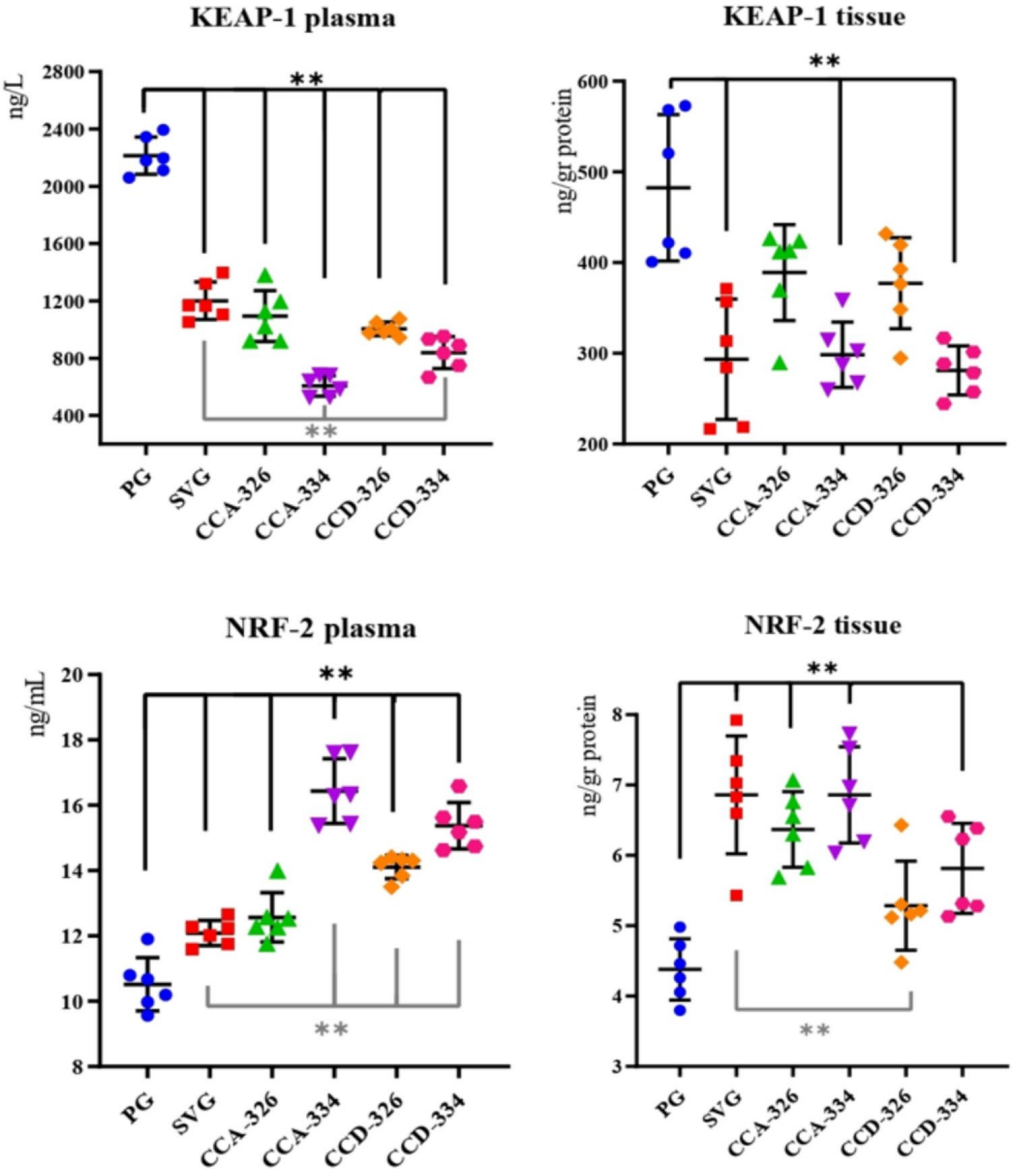
Changes in plasma and tissue KEAP-1 and NRF-2 levels by groups

Plasma and tissue KEAP1 levels were significantly reduced in CCA-326, CCA-334, CCD-326, CCD-334, and SVG in comparison to PG (*p* < 0.001). The reductions in plasma levels of KEAP1 in the CCA-334 and CCD-334 groups were also significant relative to SVG (†). The lowest tissue KEAP1 level was observed in CCD-334, and the highest was observed in PG. Plasma and tissue NRF2 levels were significantly elevated in all chalcone groups compared to PG (*p* < 0.001), except for tissue NRF2 levels in CCD-326. Plasma NRF2 levels in CCA-334, CCD-326, and CCD-334 were also significantly higher than those in SVG (†), where CCA-334 had the highest plasma levels, and CCA-334 and SVG had the highest tissue levels (Table 3, Fig. 5).

**Fig. 6:**
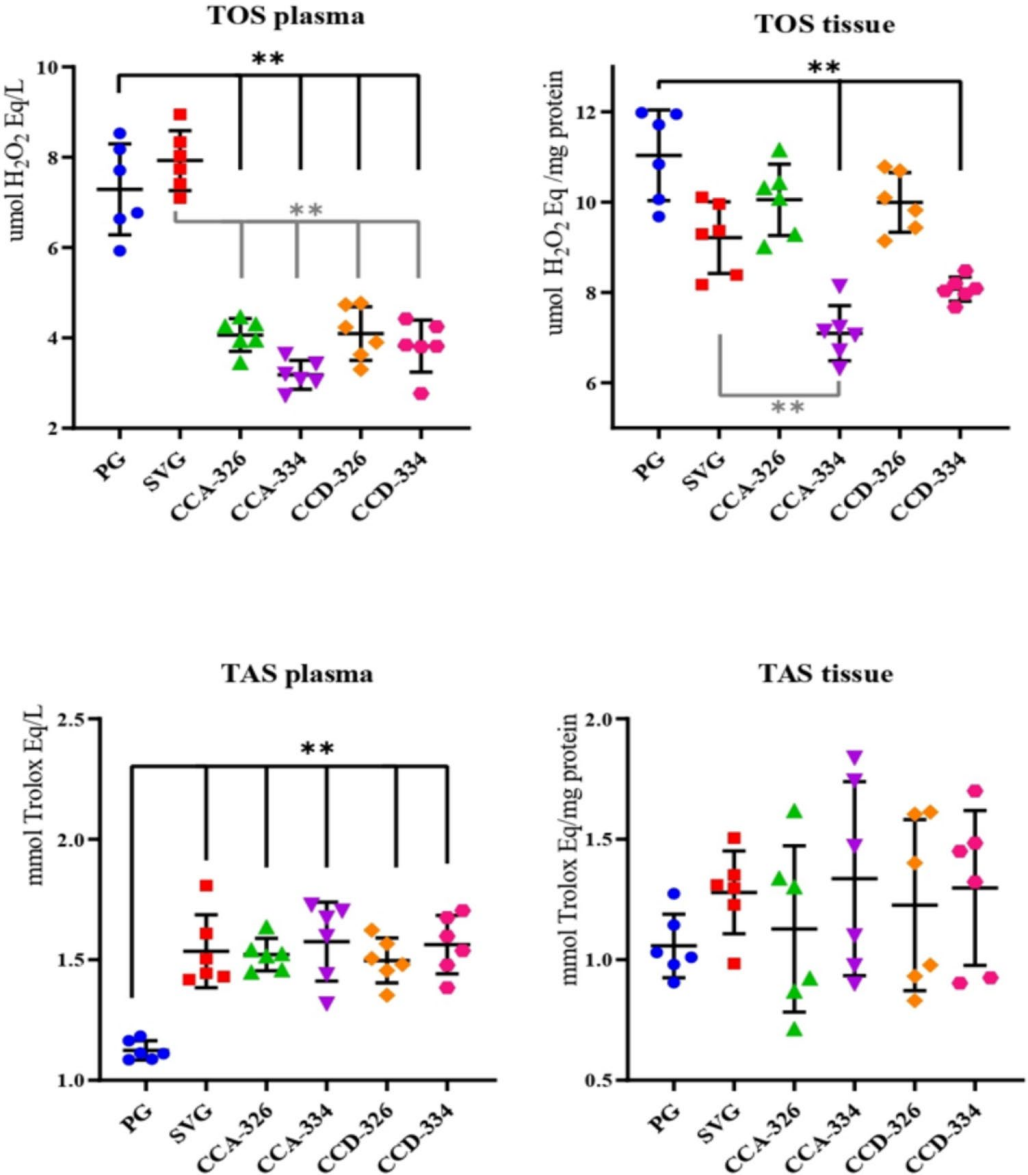
Changes in plasma and tissue TAS and TOS by groups

Plasma TAS values were significantly elevated in all chalcone groups compared to PG (*p* ≤ 0.001). Plasma TOS values were significantly reduced in the CCA-326, CCA-334, CCD-326, and CCD-334 groups compared to PG and SVG (*p* ≤ 0.001). Tissue TOS values were significantly lower in the CCA-334 and CCD-334 groups compared to PG (*p* ≤ 0.001) (Table 4, Fig. 6).

In summary, the CCA-334 and CCD-334 groups exhibited significant increases in TAS values and NRF2 levels and significant reductions in TOS values and KEAP1 levels in both plasma and tissue samples (*p* ≤ 0.001) (Tables 3 and 4, Fig. 5 and 6).

*In silico* analyses were performed to gather complementary computational information alongside the experimental findings. Blind molecular docking analyses were conducted to investigate potential binding cavities and plausible ligand orientations, and the resulting docking poses are presented in the Supplementary Material (Supplementary Figures S1 and S2). In parallel, *in silico* ADMET prediction analyses were conducted to obtain an overview of the predicted pharmacokinetic and toxicity-related properties of the synthesized compounds. The corresponding ADMET parameters are summarized in the Supplementary Material (Supplementary Table S1).

## Discussion

Clinical and experimental data indicate that neuronal hyperexcitability, OS, and neuroinflammation significantly contribute to the process of epileptogenesis, particularly in treatment-resistant epilepsy cases [26, 27]. The excessive activation of enzymes such as COX-2 and 5-LOX [28], elevated NF-κB-mediated proinflammatory responses [11], and the disruption of the KEAP1/NRF2 antioxidant defense system [29] play critical and determining roles in these pathophysiological mechanisms. Accordingly, the investigation of novel molecules with both antioxidant and anti-inflammatory properties may offer clinically significant contributions to seizure management and neuroprotective strategies [7].

The epileptic brain is highly susceptible to oxidative damage due to its elevated oxygen consumption, limited antioxidant capacity, and rich lipid contents [27]. Post-seizure disruption of the blood–brain barrier (BBB) leads to the excessive activation of inflammatory pathways such as COX-2, 5-LOX, and NF-κB, while concurrently suppressing KEAP1/NRF2-mediated antioxidant responses [26, 30]. This cyclical process plays both a triggering and reinforcing role in the onset and progression of epilepsy [27, 30], and it may also initiate secondary processes such as synaptic remodeling and pharmacoresistance. Therefore, the modulation of the post-seizure response is considered a complementary approach in antiepileptic strategies [30].

Current trends favor the development of multitarget antiepileptic agents that modulate not only neurotransmission but also inflammation and OS [3]. Natural flavonoid derivatives, thanks to their broad-spectrum biological activities, are increasingly considered promising alternatives or adjuncts to conventional antiepileptic drugs. Chalcones, classified under the flavonoid category, exhibit therapeutic potential, particularly through the NRF2-mediated antioxidant activation and the inhibition of proinflammatory enzymes such as COX-2 [7].

The activation of the KEAP1-NRF2 axis during epileptogenesis is a critical mechanism for mitigating OS-induced neuronal damage [31]. Molecules that influence the TAS/TOS balance by improving neuronal NRF2 activity are likely to have neuroprotective effects [29]. In this study, it was hypothesized that electrophilic compounds such as chalcone derivatives could activate the NRF2 signaling pathway and protect neurons against oxidative damage, an expectation supported by the observed increase in NRF2 expression in the chalcone-treated groups. Among the chalcones analyzed in this study, CCA-334 has the strongest antioxidant properties, which could be attributed to its methoxy groups and aromatic structure, and it also has the highest TAS values in both plasma and tissue samples.

Intervention in the KEAP1/NRF2 axis is considered a promising strategy for both suppressing inflammation and preserving mitochondrial function. Previous studies indicate that KEAP1 inhibition enhances NRF2 activity and reduces seizure severity in epilepsy models [6]. Under normal physiological conditions, KEAP1 retains NRF2 in the cytoplasm in an inactive state, while its inhibition facilitates the translocation of NRF2 to the nucleus and the expression of antioxidant genes [17]. The upregulation of these genes following NRF2 translocation plays a pivotal role in suppressing OS and promoting neuroprotective responses. Indeed, certain chalcone derivatives directly activate NRF2 [9, 14, 32]. In this study, the relationships between changes in the NRF2/KEAP1 axis, TAS/TOS balance, and NF-κB levels are statistically significant. The post-treatment increase in NRF2 levels supports the potential of this pathway to counteract oxidative damage [5]. As noted by Parsons et al. [4], the activation of this pathway may suppress the cascade of oxidative responses and provide neuroprotection. In this context, the chalcone derivatives used in this study, particularly CCA-334 and CCD-334, may support deficient endogenous antioxidant defenses. As emphasized by Kishore et al. [33], the reconfiguration of the KEAP1-NRF2 axis at the epileptic focus may represent a viable therapeutic strategy.

NF-κB signaling is the central modulator of proinflammatory gene expression in both acute and chronic neurological conditions such as epilepsy. On the other hand, the NRF2/KEAP1 system not only governs cellular antioxidant defense but also plays a role in suppressing NF-κB-mediated inflammatory responses [1, 2]. The crosstalk between these two pathways suggests that the simultaneous activation of NRF2 and inhibition of NF-κB may offer a dual therapeutic strategy in epilepsy cases [17].

The activation of the COX-2 and NF-κB pathways operates synergistically with oxidative responses. Playing a central role in the pathophysiology of epilepsy, NF-κB enhances free radical production and is itself reactivated by these radicals, forming a self-perpetuating cycle that sustains inflammation. In this study, the disrupted TAS/TOS balance observed alongside elevated NF-κB levels in the epilepsy group (PG) is consistent with results reported in the literature demonstrating the concurrent roles of inflammation and OS in epileptogenesis [34]. Furthermore, the reduction in antioxidant capacity (TAS) and increase in oxidant load (TOS) are consistent with the results reported by Łukawski and Czuczwar [5], who highlighted oxidative damage as a key determinant in epilepsy models.

Chalcones, thanks to their aromatic ketone backbone and conjugated double bond systems, has the capacity to bind a wide range of biological targets and modulate molecular pathways. Showing that chalcone derivatives can target inflammatory proteins such as COX-2, 5-LOX, and NF-κB, recent findings increasingly promote their utilization as anti-inflammatory and neuroprotective agents [28].

COX-2 is an inducible enzyme that is excessively expressed under inflammatory conditions and plays a central role in prostaglandin synthesis. Selective COX-2 inhibitors offer the advantage of suppressing inflammation with lower gastrointestinal toxicity when compared to classical non-selective NSAIDs, and they may reduce the frequency and severity of seizures. The activation of COX-2 in neuroinflammatory processes associated with epilepsy underscores the relevance of investigating molecules that target this enzyme for potential antiepileptic effects [35].

Vezzani et al. [27] reported that COX-2 and NF-κB activation played a central role in seizure initiation and progression, and COX-2-mediated prostaglandin E₂ (PGE₂) production increased in association with epileptic activity, making this enzyme a viable therapeutic target. The planarity, hydrogen bond donor/acceptor groups, and lipophilic side chains observed in chalcone derivatives provide high-affinity interactions with COX-2, representing key pharmacophoric characteristics. Structure–activity relationship (SAR) analyses indicate that p-methoxy and p-chloro groups on the phenyl ring improve COX-2 binding affinity and biological activity [35]. In this study, CCA-326 and CCA-334, both containing p-methoxy and p-chloro groups, have significantly lower NF-κB and COX-2 levels in plasma and tissue samples compared to other groups, highlighting the pathological roles of these inflammatory pathways in epileptogenesis.

As two distinct branches of arachidonic acid metabolism, COX synthesizes proinflammatory prostanoids, whereas 5-LOX produces leukotrienes. The use of COX inhibitors alone may redirect arachidonic acid flow toward the 5-LOX pathway, potentially leading to adverse effects. Therefore, dual inhibitors targeting both COX-2 and 5-LOX may yield a more balanced and effective anti-inflammatory response, offering therapeutic advantages in conditions accompanied by neuroinflammation. The simultaneous suppression of prostaglandin and leukotriene synthesis by dual inhibitors offers a promising strategy for managing neuroinflammatory processes such as epilepsy [15]. Minutoli et al. [36] similarly demonstrate that the inhibition of COX-2 and 5-LOX expression contributed to reduced inflammation and the preservation of neuronal integrity. In this context, the chalcone derivatives evaluated in this study seem to mimic the neuroprotective effects of COX/LOX dual inhibitors. Aroua et al. [13] report that pyrazoline derivatives exhibited high binding affinity for COX-2, and their anticonvulsant effects were associated with aromatic groups. Accordingly, the 4,5-dihydro-1H-pyrazole derivatives examined in this study may exhibit effects not only on COX-2 but also on other inflammation-related targets such as 5-LOX. These results suggest that chalcone-based pyrazoline derivatives influence multiple pharmacological targets and share similar activity profiles with pyrazole-based molecules reported to have antiepileptic properties. In particular, the suppressive effects of CCA-326 and CCA-334 on COX and LOX enzymes are consistent with the targeting strategies proposed by Vezzani et al. [27]. Thus, chalcone derivatives may offer therapeutic benefits in epilepsy by limiting the production of inflammatory lipid mediators such as PGE2.

Previous *in silico* studies suggest that certain chalcone derivatives may interact with COX-2/5-LOX enzymes as well as components of the KEAP1-NRF2 axis, potentially modulating both inflammatory and oxidative stress-related pathways [8, 28]. In line with these observations, CCA-334 exhibits a favorable predicted ADMET profile, providing supportive computational insight into its pharmacokinetic characteristics alongside the observed *in vivo* efficacy. Within this framework, the exploratory docking analyses conducted in the present study suggest that CCA-334 may plausibly engage targets involved in inflammatory processes, thereby offering a complementary computational perspective to the experimental findings.

The reductions in COX, LOX, and NF-κB levels observed with chalcone derivatives in this study are consistent with the results provided by Rabidas et al. [37], who report that flavonoids similarly inhibited inflammatory pathways. Moreover, KEAP1, NRF2, and TAS/TOS data obtained in this study corroborates with the hypothesis that the activation of the NRF2 pathway can suppress OS [37]. Among the chalcone derivatives evaluated here, CCA-334 stands out as a particularly promising candidate due to its capacity to simultaneously modulate inflammatory and OS pathways.

Even though the present study provides comprehensive in vivo biochemical evidence for the anti-inflammatory and antioxidant effects of chalcone-based pyrazoline derivatives, certain limitations should be acknowledged. Although *in silico* molecular docking and ADMET prediction analyses are included to provide supportive computational insights, these approaches remain qualitative in nature and do not replace experimental pharmacokinetic, toxicity, or direct target validation studies.

The present study does not employ direct *in vitro* or *in vivo* toxicological evaluations. However, previous studies indicate that structurally related carboxamide and carbothioamide derivatives generally exhibit low cytotoxicity within therapeutic concentration ranges [10, 35]. Moreover, the presence of halogen and methoxy substituents, which are common structural features in drug-like molecules, is not associated with inherent toxicity in related chalcone and pyrazoline derivatives [10]. While these observations, which are consistent with those reported in the literature, provide an initial safety perspective, the absence of direct toxicological assessment remains a limitation of the present work.

In addition, functional outcomes such as seizure severity scoring, behavioral or cognitive assessments, and histopathological analyses are not included, which limits the functional and anatomical interpretation of the observed molecular changes. Future studies integrating toxicological evaluation, functional assessments, histopathological analyses, and advanced computational approaches will be necessary to further clarify the therapeutic potential and translational relevance of these compounds.

## Conclusion

The results achieved in this study demonstrate that chalcone derivatives can modulate neuroinflammation and OS associated with epilepsy through multiple mechanisms. The suppressive effects observed on the COX-2 and 5-LOX enzymes, along with the regulatory roles in the NRF2/KEAP1 and NF-κB signaling pathways, indicate that these compounds can limit inflammatory processes while enhancing antioxidant defenses. Among the derivatives evaluated, CCA-334 stands out as the most promising candidate for translational applications in epilepsy treatment due to its pronounced effects on multiple pharmacological targets.

## Supplementary Information

Below is the link to the electronic supplementary material.

Supplementary file1 (JPG 59 KB)

ESM 2 Surface representation of human cyclooxygenase-2 (COX-2) illustrating the top-ranked docking pose of CCA-334 obtained from blind docking analysis. The figure shows the predicted binding cavity and the orientation of CCA-334 relative to the protein surface (PNG 1.57 MB)

High Resolution Image (TIF 1.07 MB)

ESM 3 Surface representation of human 5-lipoxygenase (5-LOX) illustrating the top-ranked docking pose of CCA-334 obtained from blind docking analysis. The figure shows the predicted binding cavity and the orientation of CCA-334 relative to the protein surface (PNG 314 KB)

High Resolution Image (TIF 3.09 MB)

## Data Availability

No datasets were generated or analysed during the current study.
