# Supplementary figures and images for: Chalcone-based Pyrazoline Derivatives as Modulators of Neuroinflammatory and Redox Pathways in Experimental Epilepsy
Source: Mol Neurobiol. 2026 Mar 18;63(1):507. doi: 10.1007/s12035-026-05795-y (PMC12999772; doi:10.1007/s12035-026-05795-y)

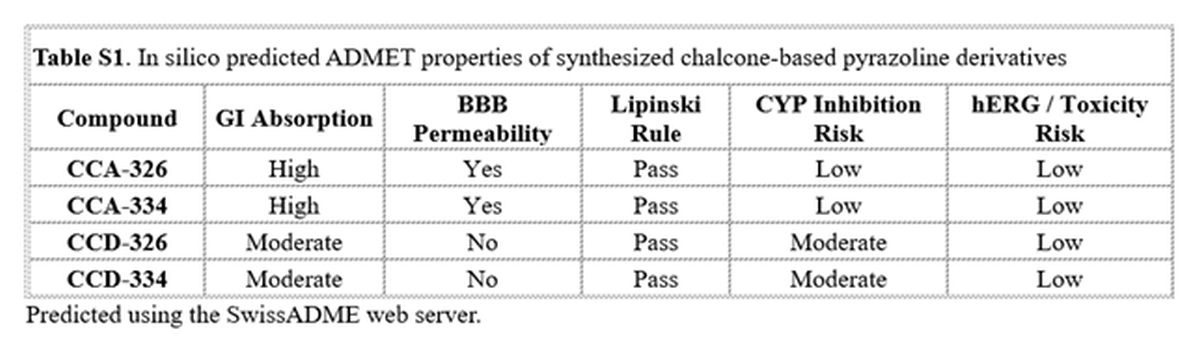

Supplement: Supplementary file 1 — Supplementary file1 (JPG 59 KB) [file 12035_2026_5795_MOESM1_ESM.jpg]

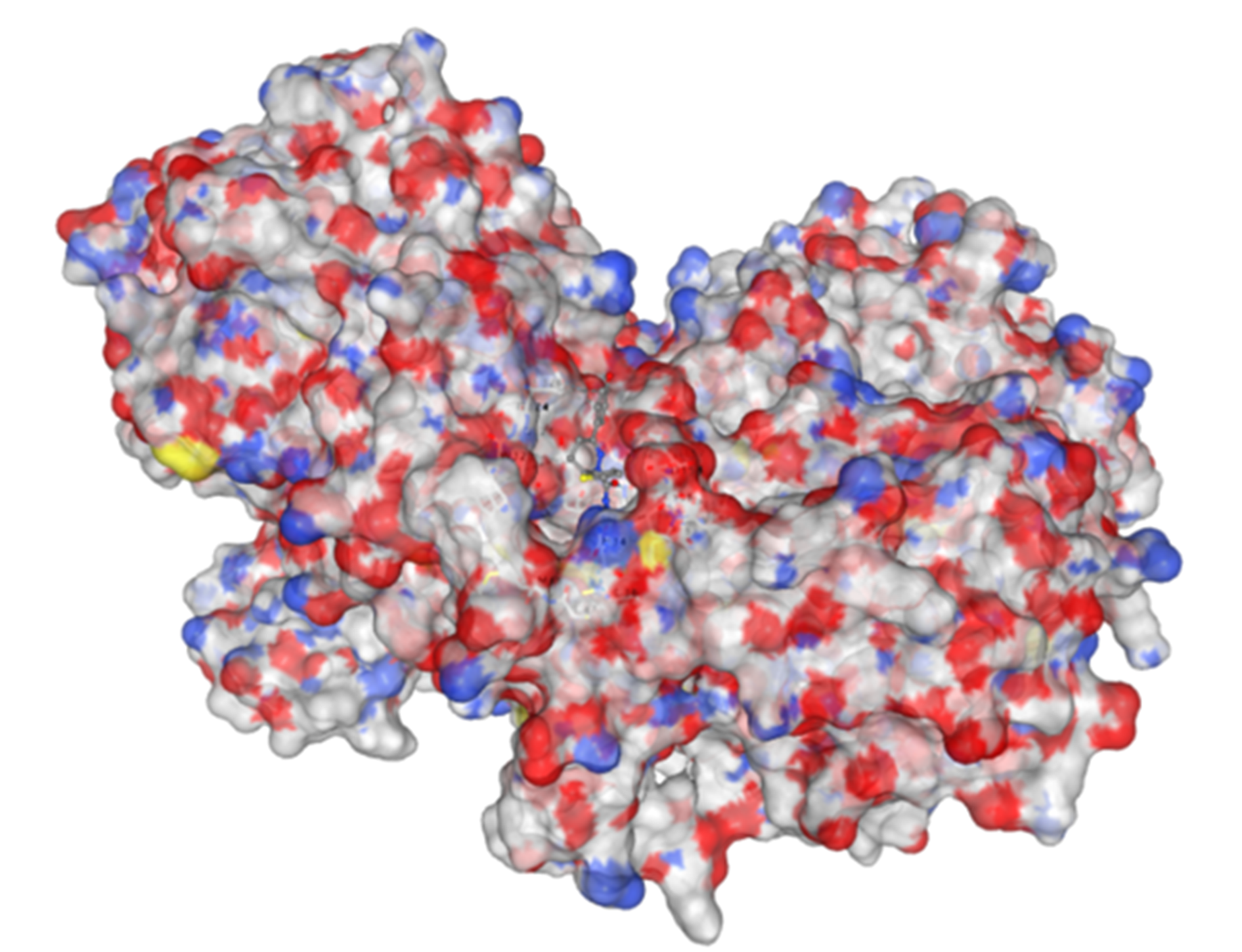

Supplement: Supplementary file 2 — Surface representation of human cyclooxygenase-2 (COX-2) illustrating the top-ranked docking pose of CCA-334 obtained from blind docking analysis. The figure shows the predicted binding cavity and the orientation of CCA-334 relative to the protein surface (PNG 1.57 MB) [file 12035_2026_5795_Fig7_ESM.png]

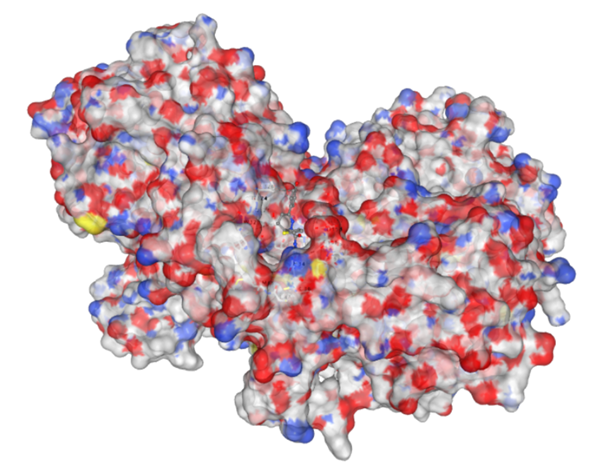

Supplement: Supplementary file 3 — High Resolution Image (TIF 1.07 MB) [file 12035_2026_5795_MOESM2_ESM.tif]

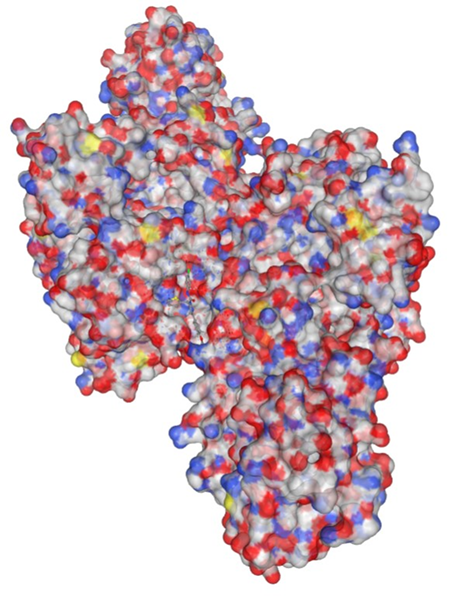

Supplement: Supplementary file 4 — Surface representation of human 5-lipoxygenase (5-LOX) illustrating the top-ranked docking pose of CCA-334 obtained from blind docking analysis. The figure shows the predicted binding cavity and the orientation of CCA-334 relative to the protein surface (PNG 314 KB) [file 12035_2026_5795_Fig8_ESM.png]

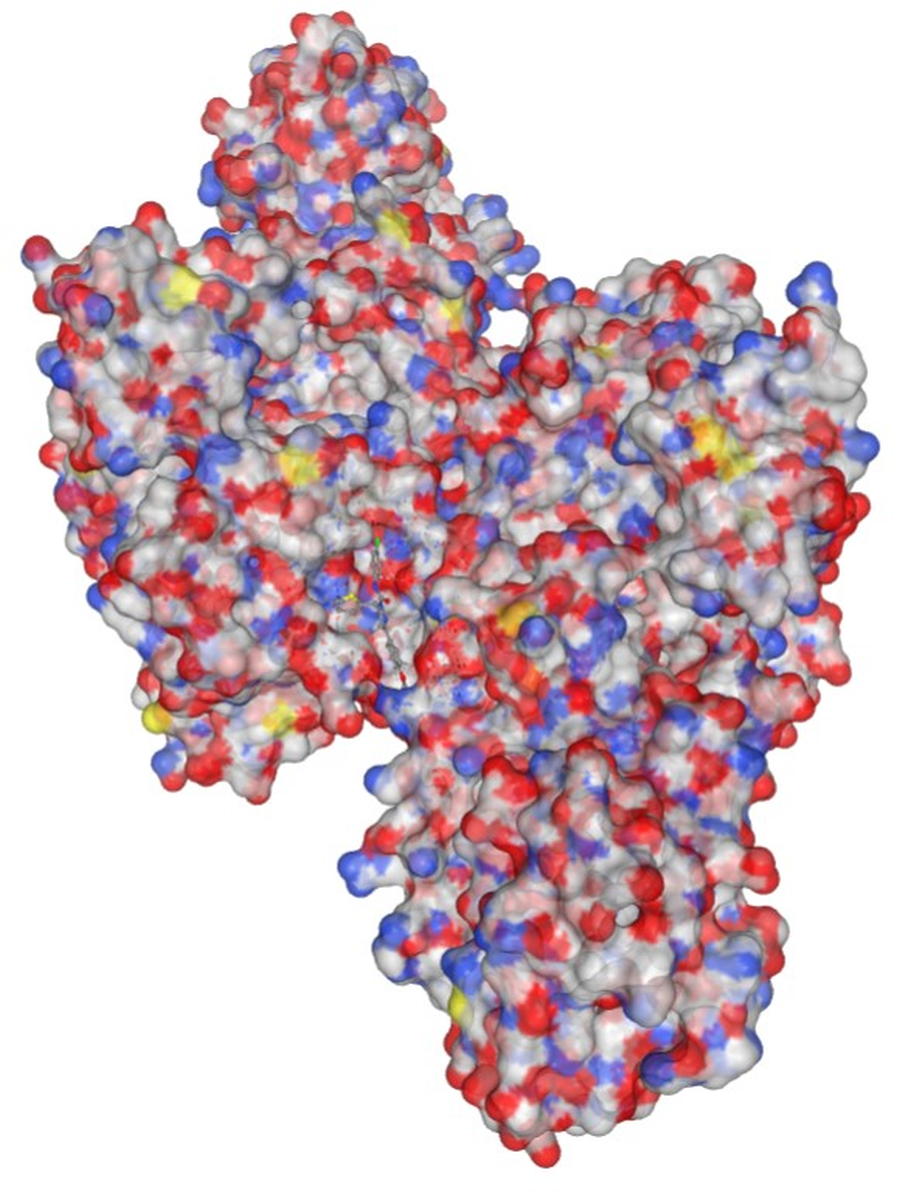

Supplement: Supplementary file 5 — High Resolution Image (TIF 3.09 MB) [file 12035_2026_5795_MOESM3_ESM.tif]
